# *Drosophila* Hrp48 Is Required for Mushroom Body Axon Growth, Branching and Guidance

**DOI:** 10.1371/journal.pone.0136610

**Published:** 2015-08-27

**Authors:** Hélène Bruckert, Giovanni Marchetti, Mirana Ramialison, Florence Besse

**Affiliations:** 1 University of Nice Sophia Antipolis, Institute of Biology Valrose, Nice, France; 2 CNRS UMR7277, Institute of Biology Valrose, Nice, France; 3 INSERM UMR1091, Institute of Biology Valrose, Nice, France; 4 Australian Regenerative Medicine Institute, Monash University, Clayton, Australia; Alexander Fleming Biomedical Sciences Research Center, GREECE

## Abstract

RNA binding proteins assemble on mRNAs to control every single step of their life cycle, from nuclear splicing to cytoplasmic localization, stabilization or translation. Consistent with an essential role of RNA binding proteins in neuronal maturation and function, mutations in this class of proteins, in particular in members of the hnRNP family, have been associated with neurological diseases. To date, however, the physiological function of hnRNPs during *in vivo* neuronal development has remained poorly explored. Here, we have investigated the role of *Drosophila* Hrp48, a fly homologue of mammalian hnRNP A2/B1, during central nervous system development. Using a combination of mutant conditions, we showed that *hrp48* is required for the formation, growth and guidance of axonal branches in Mushroom Body neurons. Furthermore, our results revealed that *hrp48* inactivation induces an overextension of Mushroom Body dorsal axonal branches, with a significantly higher penetrance in females than in males. Finally, as demonstrated by immunolocalization studies, Hrp48 is confined to Mushroom Body neuron cell bodies, where it accumulates in the cytoplasm from larval stages to adulthood. Altogether, our data provide evidence for a crucial *in vivo* role of the hnRNP Hrp48 in multiple aspects of axon guidance and branching during nervous system development. They also indicate cryptic sex differences in the development of sexually non-dimorphic neuronal structures.

## Introduction

Developing neurons extend neuronal processes—dendrites and axons—that have to navigate through a complex environment to find their targets, and establish synaptic connections. As revealed recently, neuronal cells heavily rely on post-transcriptional regulatory mechanisms such as alternative splicing, mRNA transport or precise translational control to finely tune gene expression in space and time, and regulate critical processes underlying the assembly of neuronal networks, from neurite guidance and branching to synaptogenesis [1]. Key players of these regulatory mechanisms are RNA binding proteins, which recognize specific signatures present in subsets of target mRNAs, and control their processing and cytoplasmic fate [2–4]. Consistent with a crucial role of RNA binding proteins in neural development and function, mutations in a number of these proteins have been linked to neurological disorders [5, 6]. Heterogeneous nuclear ribonucleoproteins (hnRNPs) constitute the largest family of RNA binding proteins that comprises 20 major conserved proteins designated hnRNP A to hnRNP U [7, 8]. Recently, pathogenic mutations in hnRNP A2/B1 and hnRNP A1 were found in families with inherited neurodegeneration syndroms [9]. Furthermore, members of the hnRNP A2/B1 family of proteins were identified as genetic modifiers in a fly model of Fragile X associated tremor ataxia syndrome (FXTAS) [10, 11]. Surprisingly, the physiological function of hnRNP A/B proteins during central nervous system (CNS) maturation has so far remained unclear.

To characterize the *in vivo* role of hnRNP A/B proteins during CNS maturation, we have explored the function of *Drosophila* Hrp48 (also known as Hrb27C), a fly homologue of mammalian hnRNP A2/B1 [12], using Mushroom Bodies (MBs) as a model system. MBs are symmetrical structures located in the central brain. They are essential for higher order function including olfactory learning and memory [13], and have a precisely described and stereotypic development [14]. Each MB is composed in adult of about 2,000 neurons projecting their axons ventrally toward the anterior surface of the brain, where axonal branches segregate into dorsal and medial terminal lobes. MB neurons have been subdivided into three main populations (αβ, α’β’ and γ neurons) based on their birth order, the markers they express, and the characteristic morphology of their axonal projections in the lobes [14–16]. While each αβ and α’β’ neuron bifurcates and sends one axon branch to the dorsal lobe and one to the medial lobe, adult γ neurons only project to the medial lobe.

Here, we show that Hrp48 controls multiple aspects of MB axonal development, including axon growth, branching and guidance. We have also discovered that Hrp48 is required to prevent the overextension of dorsal αβ and α’β’ axonal branches, and that the penetrance of this phenotype is much stronger in females than in males. Finally, our results indicate that Hrp48 is restricted to MB cell bodies *in vivo*, and that it is thus unlikely to mediate axonal transport and local translation of target mRNAs in this system. Together, these results highlight the biological importance of post-transcriptional regulation during CNS development. Furthermore, they reveal cryptic sex-specific differences in the regulation of MB neuron development.

## Materials and Methods

### Fly stocks

Unless specified, *Drosophila melanogaster* were grown on standard media at 25°C. l(2)02647 flies were obtained from the Bloomington Stock Center (BSC), and l(2)K16203 and l(2)K10413 flies from Anne Ephrussi laboratory [17]. These P-element insertions are referred to as *hrp48* ^*02647*^, *hrp48* ^*k16203*^ and *hrp48* ^*k10413*^, respectively, in the manuscript. The FRT40, *hrp48* ^*K02814*^ and FRT40, *hrp48* ^*7E7-18*^ chromosomes are described in [17] and [18] respectively. UAS-RNAi-*hrp48* ^*#101555*^ and UAS-RNAi-*hrp48* ^*#16041*^ were obtained from the Vienna *Drosophila* Stock Center, and the UAS-Flag-*hrp48* fly line is a gift from Tamaki Yano. UAS-mCD8-GFP and OK107-Gal4 lines were obtained from the Bloomington Stock center. MARCM clones were generated as described in [14, 19, 20].

### Immunostaining

Brains were dissected in cold PBS 1X pH 7.4, fixed in 4% formaldehyde/PBT (PBS with 0.1% Triton X-100) for 25 minutes, then washed three times 15 minutes in PBT, and incubated overnight at 4°C with 0.3% Triton X-100 and 1% BSA. Samples were then incubated overnight at 4°C with primary antibodies in PBT, washed in PBT, and incubated overnight with secondary antibodies (4°C). After three washes in PBT, samples were mounted for analysis with Zeiss LSM 510 META or Leica SPE confocal microscopes. The following primary antibodies were used: mouse anti-FasII antibody (1:15; mAb1D4 from Developmental Studies Hybridoma Bank); rabbit anti-Hrp48 antibody (1:400; gift from D. Rio); rabbit anti-GFP antibody (1:1000; A1222, Molecular Probes). Cy3- or Cy5-coupled secondary antibodies (Jackson) were used at 1:1000.

### Image analysis

Phenotypic classes (symmetric, asymmetric, truncated and ectopic projections) were defined based on FasciclinII staining. αβ projections were classified as asymmetric when the ratio of α lobe to β lobe thicknesses was significantly different from control conditions (*ie* when its gap to the mean control ratio was larger than 2 times the standard deviation). Lobe thicknesses were measured on z projections of stacks of confocal images using the Zeiss LSM Image Browser or the Leica LAS AF Lite softwares. As α lobe diameter varies along the dorsoventral axis, we focused on α lobe central most-region and defined α lobe width as the largest diameter in this region. α or β lobes were classified as truncated when they did not fully extend or did not develop at all. Ectopic projection defects were counted separately, as they cannot be quantified on truncated lobes and can be detected on asymmetric lobes.

The number of MB neurons was counted on single confocal sections of mCD8-GFP-expressing neurons. Sections to be analysed were chosen such that the cell bodies of all peripheral-most MB neurons were visible. Non-properly oriented brains were not considered for the analysis.

### Western Blot

Western blots were performed using standard procedure, and the equivalent of 8 adult brains was loaded per lane. The following antibodies were used: rabbit anti-Hrp48 (1:2500; gift from D. Rio); mouse anti-Tubulin (1:10000; Sigma); IR-Dye 800 anti-rabbit 800 (1:10000; Li-Cor); Alexa 680-anti-mouse (1:10000; Molecular probes). Signals were revealed using the Li-cor Odyssey imaging system and quantified using ImageJ.

## Results

### Hrp48 is required for the growth, guidance and branching of MB αβ and α’β’ axons

**Fig 1:**
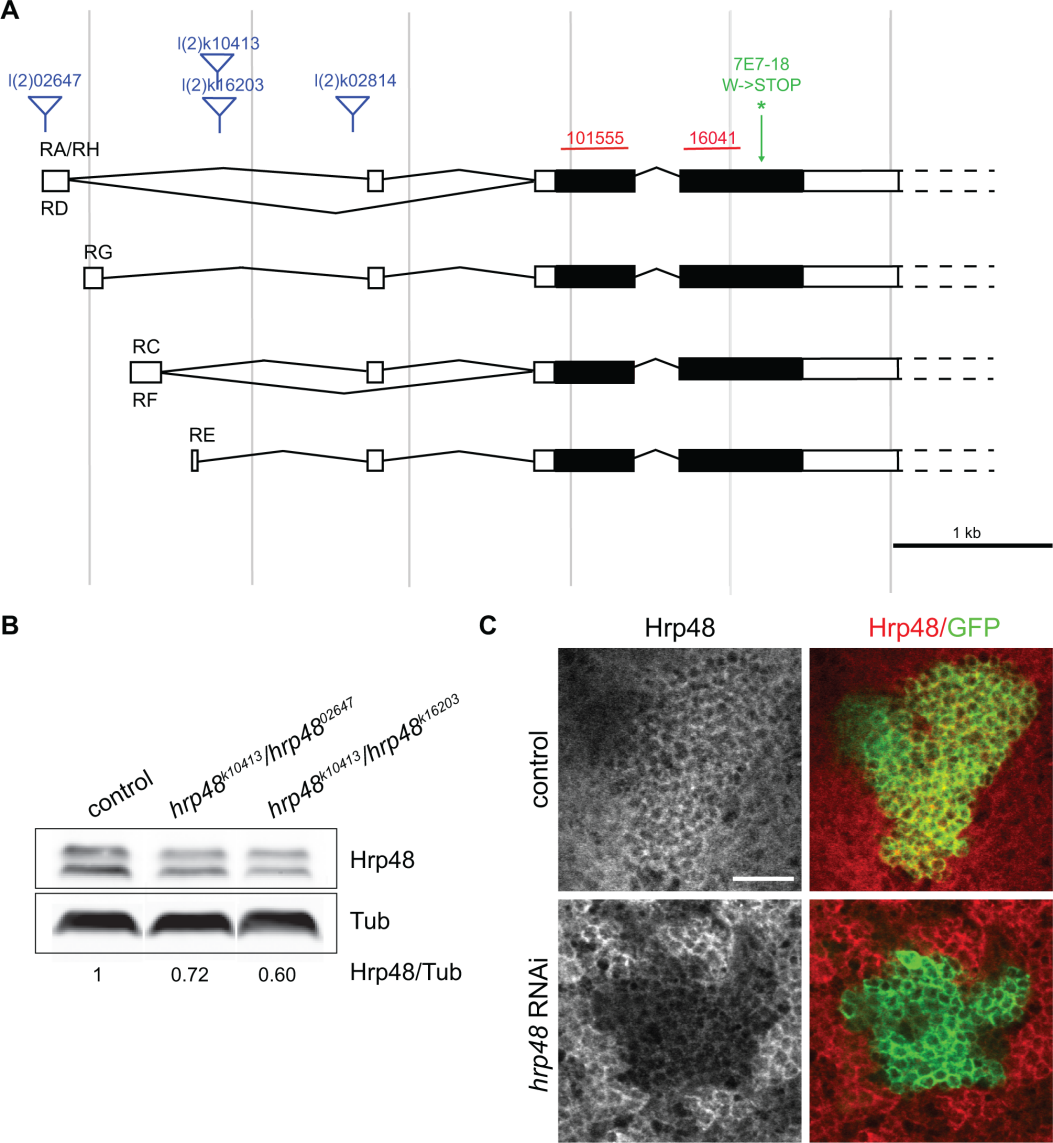
*hrp48* locus and mutants. (A) Genomic organization of *hrp48* locus, and intron-exon structure of *hrp48* transcripts. Untranslated and coding regions are represented respectively as white and black boxes. Positions of the P-elements inserted into the l(2)02647, l(2)k16203, l(2)k10413 and l(2)k02814 strains are shown in blue. Sequences targeted by the RNAi lines #101555 and 16041 are represented in red. Position of the EMS point mutation found in the 7E17-8 line is indicated in green. This mutation generates a premature stop codon at position 312 (http://flybase.org/reports/FBrf0198712.html). Note that the transcript nomenclature follows that of Flybase (FB2015_01 release). For the sake of simplicity, alternative 3’UTRs are not represented in the scheme (see open dotted boxes). (B) Western-Blot of control (w) and mutant (*hrp48* ^*k10413*^/*hrp48* ^*02647*^ and *hrp48* ^*k10413*^/*hrp48* ^*k16203*^) adult brain extracts probed with anti-Hrp48 (upper lane) and anti-Tubulin (Tub; lower lane) antibodies. Values shown at the bottom correspond to normalized Hrp48/Tubulin signal intensity ratios. (C) UAS-mCD8-GFP/+;;OK107-Gal4/+ (upper panel) and UAS-mCD8-GFP/UAS*-RNAi*-*hrp48* ^#101555^;;OK107-Gal4/+ (lower panel) larval brains stained with anti-Hrp48 antibodies (left, red in the overlay) and GFP (green in the overlay). Note the reduced Hrp48 levels observed in MB cell bodies upon RNAi expression. Scale bar: 10 μm.

Hrp48 is an essential gene whose function is required for embryonic development [21]. To investigate the function of *hrp48* in MB morphogenesis, we thus first used two semilethal transheterozygous combinations of *hrp48* mutant alleles—*hrp48* ^*k10413*^/*hrp48* ^*02647*^ and *hrp48* ^*k10413*^ */hrp48* ^*k16203*^ [21]—and two inducible RNAi lines targeting different regions of *hrp48* coding sequence—#16041 and #101555 - (Fig 1A). These mutant conditions were associated with a partial, but significant decrease in Hrp48 levels, as visualized by Western-Blot analysis on *hrp48* ^*k10413*^/*hrp48* ^*02647*^ and *hrp48* ^*k10413*^ */hrp48* ^*k16203*^ adult brain lysates (Fig 1B), and by anti-Hrp48 immunostaining on flies expressing *hrp48* RNAi constructs in MB neurons (Fig 1C).

**Fig 2:**
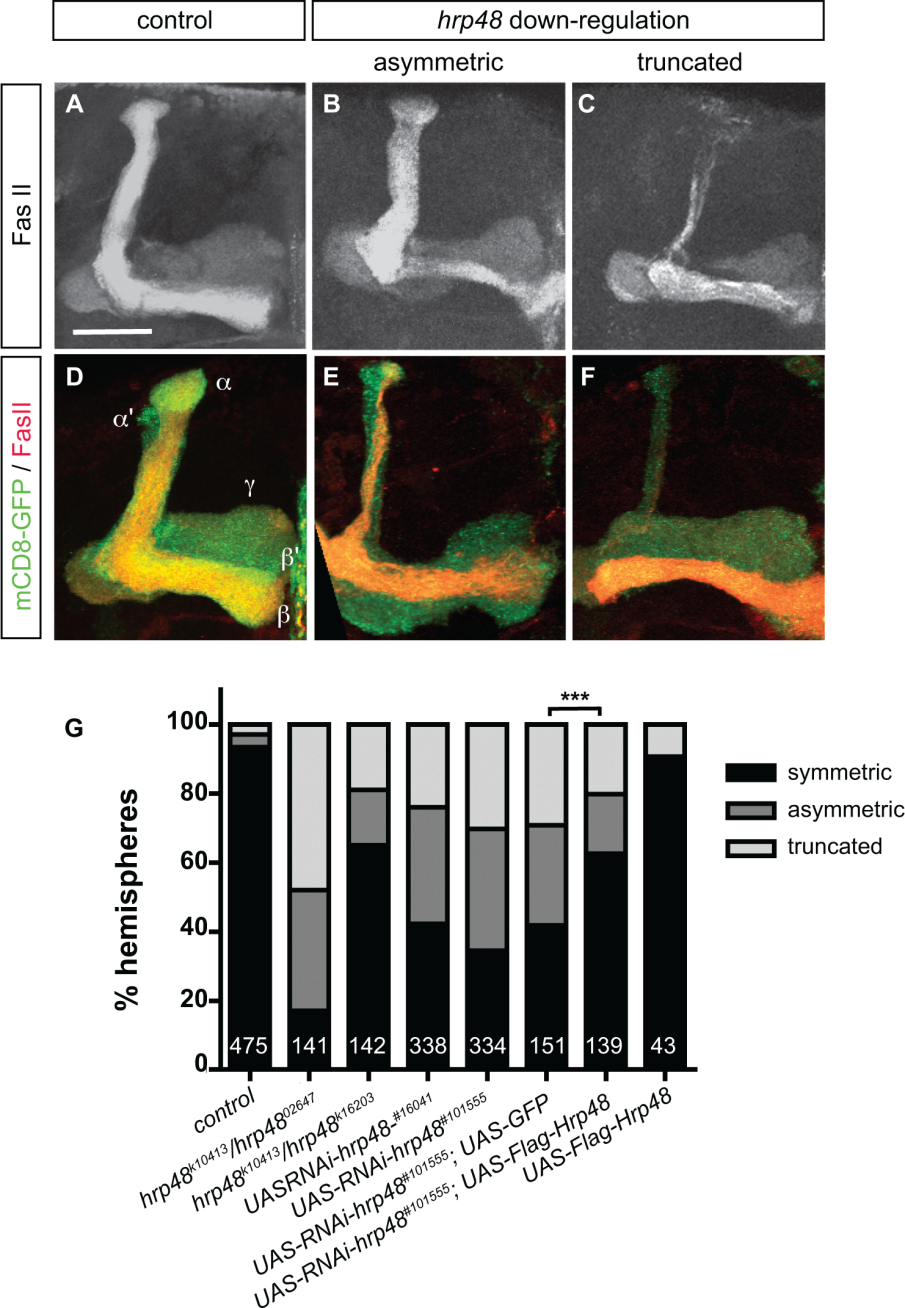
*hrp48* downregulation results in MB lobe projection defects. (A-C) Mushroom Body (MB) lobes of control (A), *hrp48* ^*k10413*^/*hrp48* ^*k16203*^ (B), or *hrp48* ^*k10413*^/*hrp48* ^*02647*^ (C) adult brains stained with anti-FasciclinII (FasII) antibodies. FasII is strongly expressed in αβ neurons, weakly in γ neurons. (D-F) MB lobes of control (D) or *hrp48*-RNAi (E,F) adult brains expressing mCD8-GFP (green), and stained with anti-FasII antibodies (red). Representative examples of the asymmetric and truncated categories used for quantification are shown. Precise genotypes: UAS-mCD8-GFP/+;;OK107-Gal4/+ (D); UAS-mCD8-GFP/UAS-RNAi-*hrp48* ^*#101555*^;;OK107-Gal4/+ (E,F); UAS-mCD8-GFP/+;UAS-RNAi-*hrp48* ^*#16041*^ */+*;OK107-Gal4/+ (G). Scale bar in A-F: 20μm. (G) Percentages of MBs exhibiting symmetric projections, asymmetric projections or truncated lobes (either α or β) in controls (*w*), *hrp48* transheterozygous combinations, and two independent RNAi lines (#16041 and #101555). Two controls were used for the rescue assay: UAS-mCD8-GFP/UAS-RNAi-*hrp48* ^*#101555*^;UAS-GFP/+;OK107-Gal4/+ flies as a control of Gal4 titration, and UAS-mCD8-GFP/+;UAS-Flag-Hrp48/+;OK107-Gal4/+ flies to ensure that Hrp48 overexpression did not induce axon projection defects. Numbers represent numbers of scored hemispheres. Statistical comparison to the UAS-GFP control: ***, p<0.001 (χ^2^ test).

To visualize the morphology of adult MB lobes, we used FasciclinII (FasII) antibodies that weakly label γ axons and strongly label αβ axons. Furthermore, a membrane-tagged form of GFP (mCD8-GFP) was introduced in RNAi contexts to visualize the axonal projections of the entire population of MB neurons. As shown in Fig 2A–2F, a general decrease in MB lobe volume associated with a decrease in the total number of MB neurons (S1A Fig) was observed upon *hrp48* downregulation, suggesting that Hrp48 may play a role in the regulation of MB neuroblast proliferation.

Most strikingly, defects in MB αβ axon projection patterns were observed in *hrp48* mutants. As revealed by anti-FasII staining, αβ neurons normally produce two branches projecting dorsally and medially to form dorsal and medial lobes of similar width (Fig 2A). In *hrp48* ^k10413/^ *hrp48* ^*02647*^ and *hrp48* ^*k10413/*^ *hrp48* ^*k16203*^ animals, in contrast, MBs with α and β lobes of unequal width (asymmetric, Fig 2B and 2G) were observed. In addition, MBs with truncated α lobes (Fig 2C), or MBs lacking either the α or the β lobe (Fig 2G and data not shown) were frequently observed. These defects reflect a MB-autonomous function of *hrp48* in MB axon morphogenesis, as expressing UAS-RNAi constructs under the control of the MB-specific OK107-Gal4 driver produced similar αβ lobe defects, including asymmetric, shorter or missing lobes (Fig 2D–2F and 2G). As expected from Gal4/UAS-driven phenotypes, axonal projection defects were stronger at 29°C than at 25°C (S1B Fig). Furthermore, they were partially, but significantly suppressed upon expression of a wild-type copy of *hrp48* (Fig 2G), confirming that they indeed result from a downregulation of *hrp48*.

**Fig 3:**
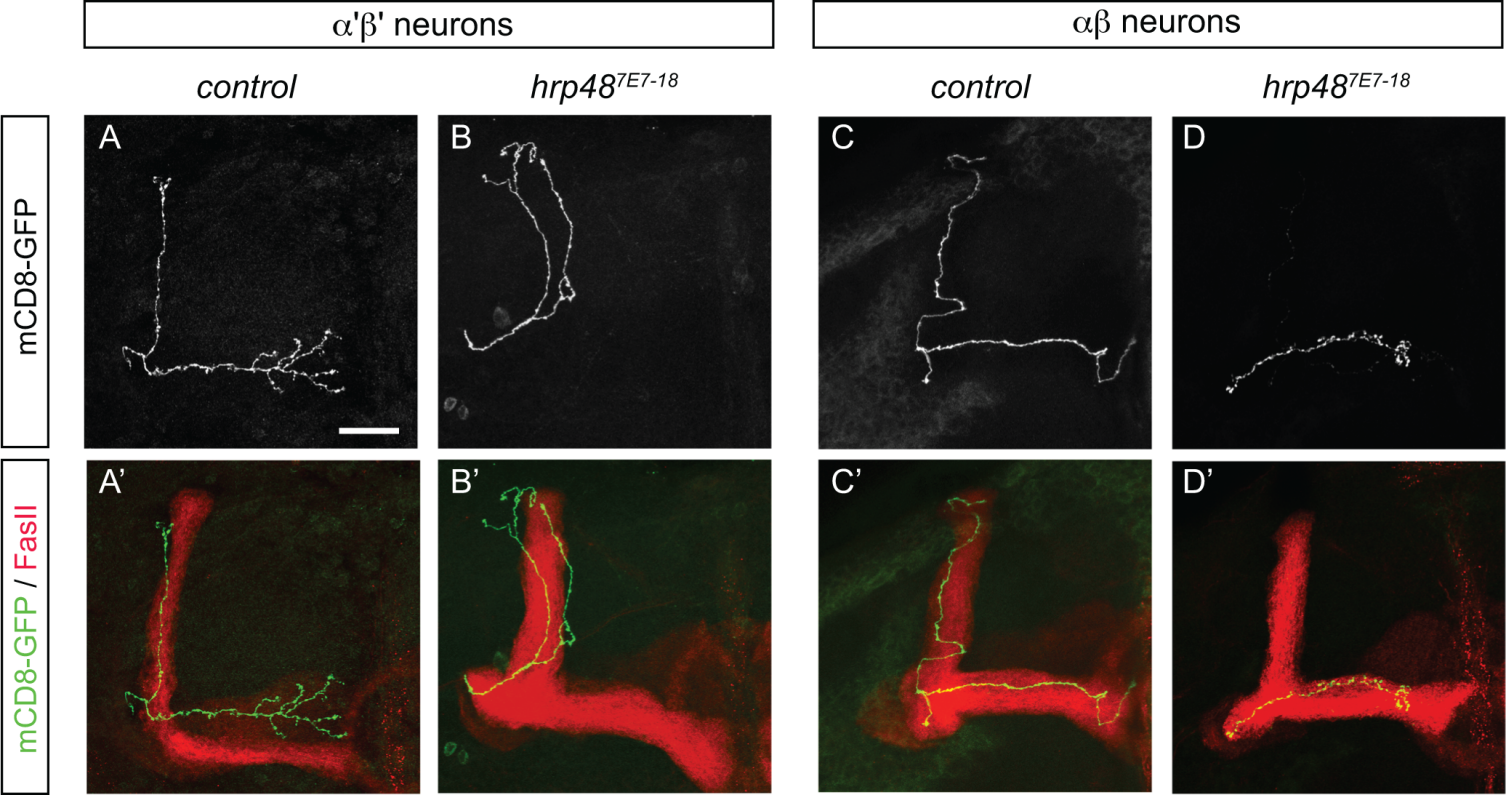
*hrp48* is required for axonal growth, guidance and branching. (A,B) Axonal projections of single wild-type (A) and *hrp48* ^*7E7-18*^ (B) adult α’β’ neurons generated using the MARCM technique. (C,D) Axonal projections of single wild-type (C) and *hrp48* ^*7E7-18*^ (D) adult αβ neurons generated using the MARCM technique. Projections are labeled by mCD8-GFP (white in A-D; green in A’-D’) and FasII (red in A’-D’). Note that the αβ neuron shown in D exhibits both branching and growth defects. Precise genotypes: hsp-flp, UAS-mCD8-GFP/+; FRT40+/FRT40A tub-Gal80;; OK107-Gal4/+ (A,C) and hsp-flp, UAS-mCD8-GFP/+; FRT40A *hrp48* ^*7E7-18*^/FRT40A tub-Gal80;; OK107-Gal4/+ (B,D). Scale bar: 20μm.

While the truncated lobe phenotype reflects a role of Hrp48 in axon growth, the uneven distribution of αβ processes between the dorsal and the medial lobes may either result from a failure of αβ neurons to form two independent branches (branching defects), or from defects in segregating axonal branches in divergent directions (guidance defects). To discriminate between these hypotheses and precisely visualize the morphology of both αβ and α’β’ *hrp48* mutant neurons, we generated single αβ or α’β’ neurons homozygous for the lethal mutation 7E7-18 (Fig 1A, [18]) using the MARCM technique [14, 20]. *hrp48* ^*7E7-18*^ αβ or α’β’ mutant neurons exhibiting two branches projecting in the same lobe were observed (Fig 3A and 3B and data not shown). Furthermore, *hrp48* ^*7E7-18*^ αβ or α’β’ mutant neurons lacking a branch were observed (Fig 3C and 3D and data not shown), indicating that *hrp48* function is required both for the formation of main axonal branches, and for their guidance and growth in their respective lobes. The penetrance of these defects was however low (9/63 for α’β’ neurons and 2/26 for αβ neurons; see S1 Table for a detailed distribution of phenotypic classes), which may reflect the fact that neurons do not regulate branch formation and guidance cell-autonomously, bur rather collectively, at the population level [22].

Altogether, these results indicate that *hrp48* has pleiotropic functions and is required for axonal growth, guidance and branching of MB α’β’ and αβ neurons. Notably, *hrp48* function appears to be cell-specific, as *hrp48* mutant MB γ lobes were indistinguishable from wild-type γ lobes (Fig 2).

### Hrp48 prevents overextension of MB dorsal axonal branches

**Fig 4:**
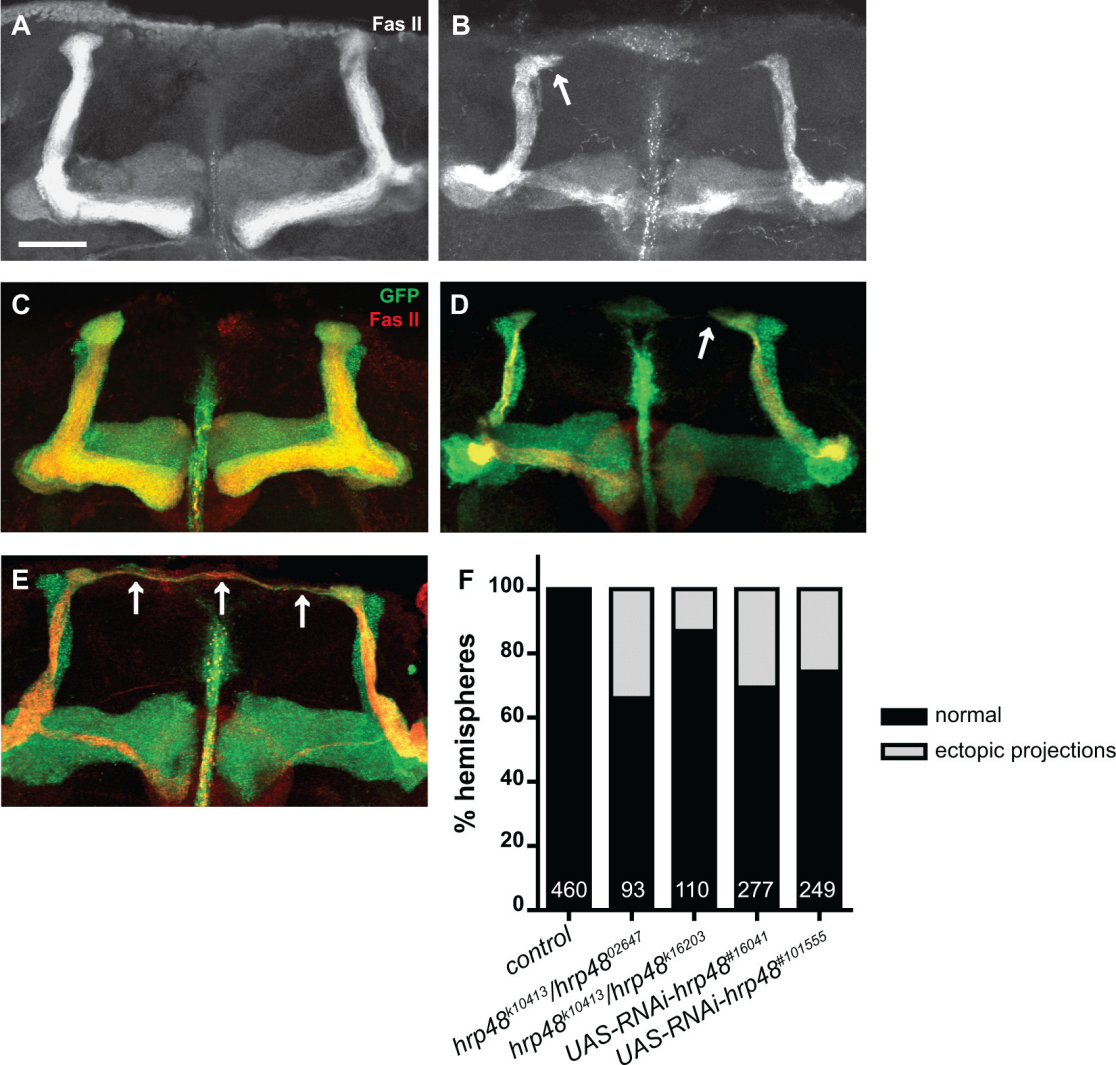
*hrp48* downregulation induces ectopic projection of MB dorsal lobes. (A,B). MB lobes of control (A) and *hrp48* ^*k10413*^/*hrp48* ^*02647*^ (B) adult brains stained with anti-FasII antibodies. (C-E) MB lobes of control (C) or *hrp48*-RNAi (D,E) adult brains expressing mCD8-GFP (green), and stained with anti-FasII antibodies (red). Arrows in B,D,E point to overextended dorsal axonal branches. Precise genotypes: UAS-mCD8-GFP/+;;OK107-Gal4/+ (C); UAS-mCD8-GFP/UAS-RNAi-*hrp48* ^*#101555*^;;OK107-Gal4/+ (D,E). Scale bar in A-E: 20μm. (F) Percentages of MBs exhibiting normal projections (*ie* dorsal branches stopping at the end of the dorsal lobe), or ectopic projections (as judged based on the αβ axon bundle).

Our analysis of *hrp48* mutant phenotypes revealed that *hrp48* MBs show an additional phenotype unrelated to defective branching or growth inhibition, and characterized by an abnormal overextension of dorsal lobes. This phenotype was observed in *hrp48* semilethal transheterozygous conditions (Fig 4B, arrow, compare with 4A; Fig 4F), as well as upon downregulation of *hrp48* specifically in MB neurons using two independent RNAi lines (Fig 4D, arrow, compare with 4C; Fig 4F). Notably, both αβ neurons (labelled by FasII, see Fig 4B and 4D) and α’β’ neurons (labelled by Trio, see S2 Fig) exhibited overextended dorsal branches. Furthermore, « overextended » dorsal branches systematically grew toward the midline, where, in extreme cases, they met with their counterparts (Fig 4E, arrows).

**Fig 5:**
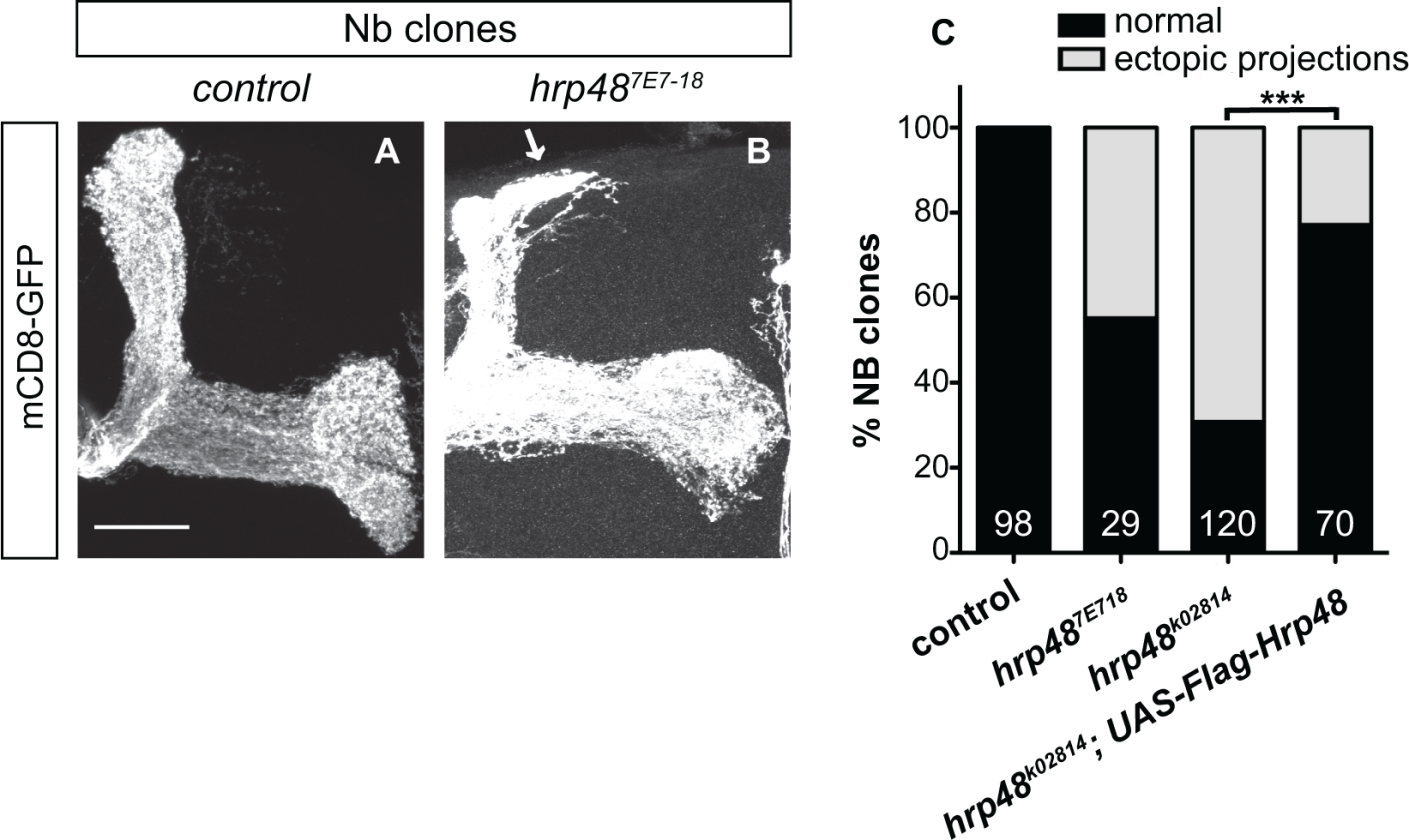
*hrp48* is required to prevent overextension of MB dorsal axonal branches. (A, B) Axonal projections of wild-type (A) or *hrp48* ^*7E7-18*^ (B) neuroblast (Nb) clones labelled by mCD8-GFP and generated using the MARCM technique. Arrow in B points to ectopically projecting axonal branches. Scale bar: 20μm. (C) Percentages of Nb clones exhibiting « normal » projections (*ie* dorsal branches stopping at the end of the dorsal lobe), or ectopic projections. Numbers represent numbers of scored Nb clones. Re-expression of a wild-type copy of Hrp48 (Flag-Hrp48) rescues the MARCM mutant phenotypes (***, p<0.001 in a χ^2^ test). Precise genotypes: hsp-flp, UAS-mCD8-GFP/+; FRT40A/FRT40A tub-Gal80;;OK107-Gal4/+ (A); hsp-flp, UAS-mCD8-GFP/+; FRT40A, *hrp48* ^*7E7-18*^/ FRT40A tub-Gal80;;OK107-Gal4/+ (B).

To confirm these phenotypes using independent lethal mutations, we generated MB neuroblast clones labelled by mCD8-GFP and composed of neurons homozygous for the *hrp48* ^*7E7-18*^ or *hrp48* ^*k02814*^ mutant alleles. As shown in Fig 5A–5C, ectopic extension of dorsal bundles was observed in about 55% and 70% of *hrp48* ^*7E7-18*^ or *hrp48* ^*k02814*^ mutant clones respectively. These defects were significantly suppressed when expressing a wild-type copy of *hrp48* in mutant neurons (Fig 5C). However, they were rarely observed in single cell-clones (2/63 *hrp48* ^*7E7-18*^ α’β’ clones and 1/26 *hrp48* ^*7E7-18*^ αβ clones; see S1 Table), suggesting that they reflect a population-autonomous rather than a strictly cell-autonomous function of *hrp48*.

Altogether, these results indicate that Hrp48 is required to prevent the overextension of MB dorsal axonal branches.

### The penetrance of *hrp48* axon overextension phenotypes is significantly higher in females than in males

**Fig 6:**
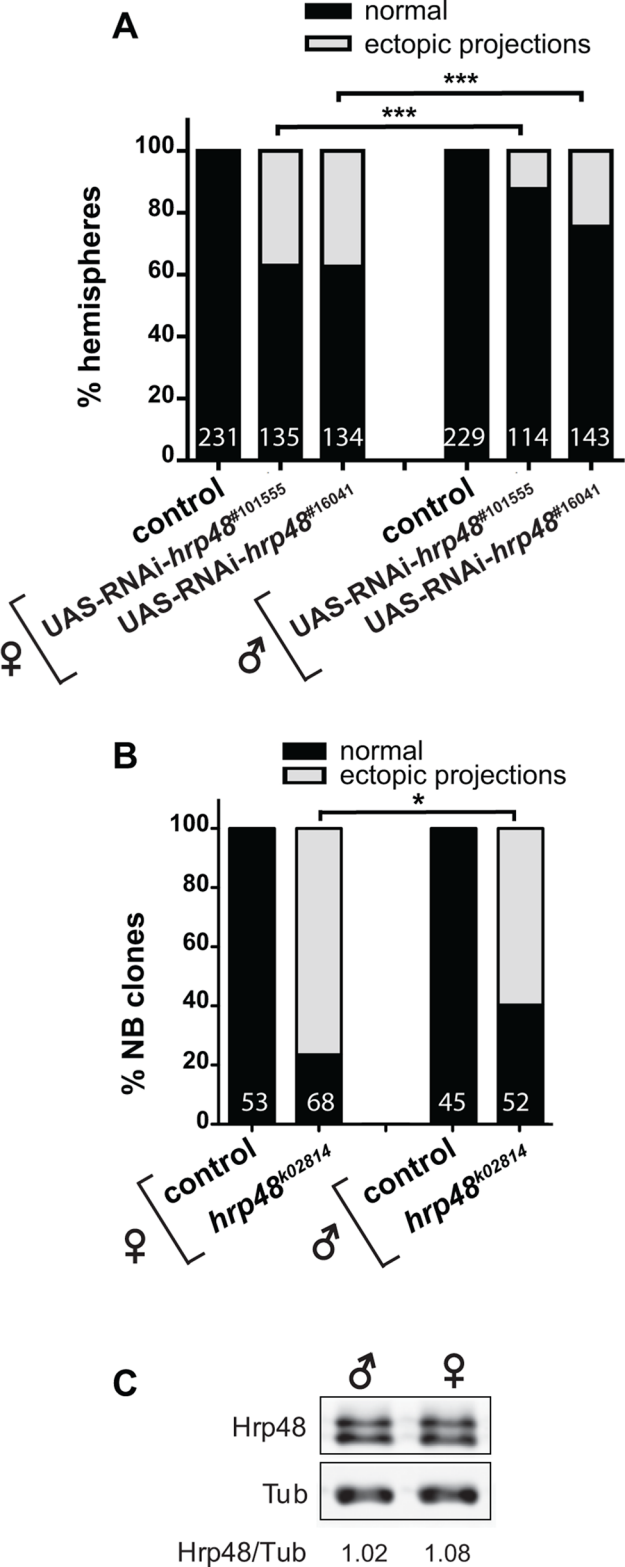
*hrp48* axon overextension defects are more penetrant in females than in males. (A) Percentages of MBs exhibiting normal projections (*i*.*e*. dorsal branches stopping at the end of the dorsal lobe), or ectopic projections in *hrp48* RNAi females (left) or males (right). Precise genotypes: UAS-mCD8-GFP/+;;OK107-Gal4/+ (+); UAS-mCD8-GFP/UAS-RNAi-*hrp48*;;OK107-Gal4/+ (*hrp48* RNAi). Numbers represent numbers of scored hemispheres. The penetrance of the ectopic projection phenotype is significantly higher in females than in males (statistical comparison to females: ***, p<0.001 in a χ^2^ test). (B) Percentages of *hrp48* ^*k02814*^ neuroblast clones exhibiting normal, or ectopic projections. Numbers represent numbers of scored Nb clones. Statistical comparison to the female context: *, p<0.05 (χ^2^ test). Precise genotypes: hsp-flp, UAS-mCD8-GFP/+; FRT40A+/FRT40A tub-Gal80;; OK107-Gal4/+ (control); hsp-flp, UAS-mCD8-GFP/+; FRT40A, *hrp48* ^*k02814*^/ FRT40A tub-Gal80;; OK107-Gal4/+ (*hrp48* ^*k02814*^). (C) Western-Blot of *w* male (left) and female (right) adult brain extracts probed with anti-Hrp48 (upper lane) and anti-Tubulin (Tub; lower lane) antibodies. Values shown at the bottom correspond to normalized Hrp48/Tubulin signal intensity ratios.

Inactivation of *hrp48* function in *Drosophila* imaginal wing discs was shown to induce aberrant wing phenotypes, with a higher penetrance in females than in males [23]. To test whether the penetrance of *hrp48* MB axon projection defects was sex-dependent, we compared the proportion of MBs with defective axonal projection patterns in males and females expressing *hrp48* RNAi construct under the control of the OK107-Gal4 driver. While the proportion of MBs with asymmetric or truncated α or β lobes was only slightly higher in *hrp48* RNAi females than in *hrp48* RNAi males (S3 Fig), the proportion of MBs with axon overextension defects was much higher in *hrp48* RNAi mutant females than in *hrp48* RNAi mutant males (Fig 6A). Notably, such a difference between males and females was observed for both UAS-RNAi lines, and upon generation of neuroblast clones mutant for the lethal *hrp48* ^*k02814*^ allele (Figs 1A and 6B).

To test whether the observed sex-specific response was due to differences in basal levels of Hrp48 in males *vs* females, we quantitatively compared Hrp48 protein levels in male and female brains. As shown in Fig 6C, no significant difference could be observed between sexes.

Thus, these results reveal a differential sensitivity of male and female MB neurons to the inactivation of *hrp48*, and cryptic differences between male and female MB development.

### Hrp48 accumulates in the cytoplasm of neuronal cell bodies at steady state

Members of the hnRNP family are multi-functional proteins that are dynamically shuttling between the nucleus and the cytoplasm and regulate both nuclear and cytoplasmic events, ranging from RNA transcription and splicing to RNA stability, transport or translational control [7]. In cultured mammalian oligodendrocytes and neurons, hnRNP A2 localizes to cellular processes and regulates the transport and the translation of target mRNAs important for different aspects of neural development [24–27]. In the polarized *Drosophila* oocyte, Hrp48 was shown to associate with the asymmetrically localized *gurken* and *oskar* mRNAs to promote their targeting to the antero-dorsal and posterior poles respectively, and to prevent their premature translation [17, 18, 28].

**Fig 7:**
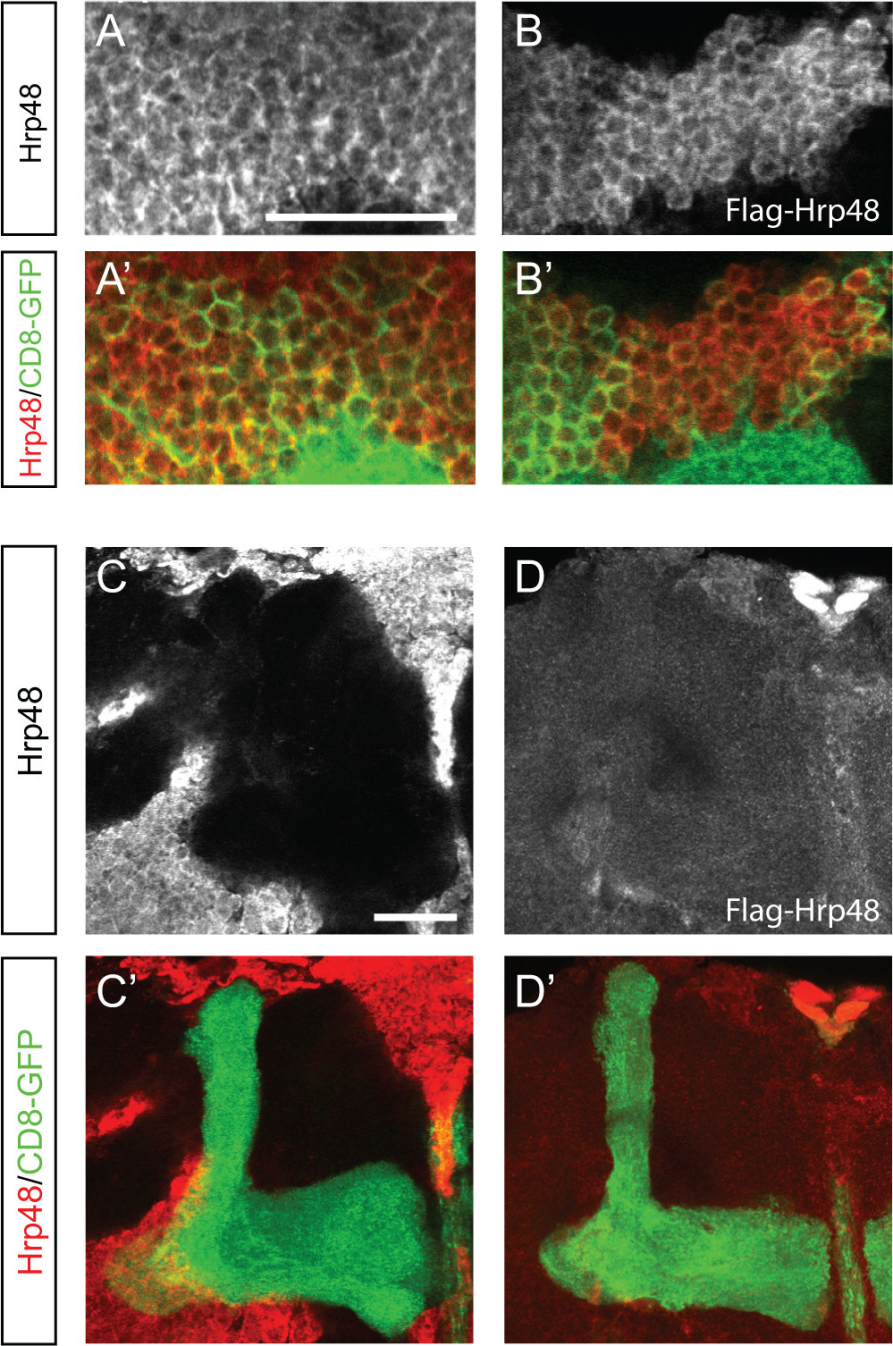
Subcellular distribution of Hrp48 in MB neurons. (A) Cell bodies of UAS-mCD8-GFP/+;;OK107-Gal4/+ adult (A) brains stained with anti-Hrp48 antibodies (white in A; red in A’) and GFP (green in A’). (B) Cell bodies of UAS-mCD8-GFP/+;UAS-Flag-Hrp48/+;OK107-Gal4/+ adult brain stained with anti-Flag antibodies (white in B; red in B’) and GFP (green in B’). Scale bar: 20μm. (C) MB lobes of UAS-mCD8-GFP/+;;OK107-Gal4/+ adult brains stained with anti-Hrp48 antibodies (white in C; red in C’) and GFP (green in C’). (D) MB lobe of UAS-mCD8-GFP/+;UAS-Flag-Hrp48/+;OK107-Gal4/+ adult brain stained with anti-Flag antibodies (white in D; red in D’) and GFP (green in D’). Scale bar: 20μm.

To determine the subcellular distribution of Hrp48 in MB neurons, we stained *Drosophila* brains with anti-Hrp48 antibodies. A cytoplasmic signal, whose intensity significantly decreased upon *hrp48* downregulation, was observed in adult and larval MB cell bodies (Figs 1C and 7A). Such a cytoplasmic accumulation was also observed when expressing a Flag-tagged version of Hrp48 in MB neurons using the OK107-Gal4 line (Fig 7B and data not shown). To test if Hrp48 was transported to MB axons, we analysed the distribution of the protein in MB lobes. No signal was detected for either endogenous Hrp48 (Fig 7C) or Flag-tagged Hrp48 (Fig 7D).

Thus, these results indicate that Hrp48 exhibits a predominantly cytoplasmic localization at steady-state. Furthermore, Hrp48 is confined to the cell bodies of MB neurons, and appears not to be targeted to axons.

## Discussion

### Hrp48 controls multiple aspects of axonal development

Members of the hnRNP A/B family of proteins have been shown to regulate the spatio-temporal expression pattern of transcripts important for neural development [25–27], yet the role of these proteins during nervous system maturation *in vivo* has remained elusive. Here, we have shown that the hnRNP A/B family member Hrp48 controls multiple aspects of axon morphogenesis in *Drosophila* brains. First, MB α’β’ and αβ neurons mutant for *hrp48* frequently exhibit shorter axons, suggesting that Hrp48 promotes the growth of axonal branches in these populations. Second, as revealed by our clonal analysis, α’β’ and αβ neurons lacking a dorsal or a medial branch, or neurons with two axonal branches projecting in the same lobe are generated upon inactivation of *hrp48* function. This indicates that Hrp48 regulates axonal branching and guidance. Finally, Hrp48 prevents ectopic axonal growth, as an overextension of dorsal MB axonal branches is observed upon *hrp48* inactivation.

Such a pleiotropic phenotype likely results from the misregulation of various mRNA targets. Although hnRNP A/B family members such as hnRNP A2 were shown to regulate the transport and the translation of target mRNAs to neurites [24–27], our immunolocalization analysis rather points to a role of Hrp48 in MB cell bodies. Given the known function of hnRNP proteins [7], this role may include translational control of target mRNAs and/or regulation of their alternative splicing. Consistent with this latter hypothesis, a systematic microarray-based analysis has revealed that alternative splicing of more than 300 transcripts is under the control of Hrp48 in *Drosophila* S2 cells [29]. Remarkably, genes involved in neuronal development and cell morphogenesis were shown to be significantly overrepresented in this population of *hrp48*-affected genes. Among interesting *hrp48* target genes identified in this study are *enable*, *trio* and *short-stop*, which encode regulators of the cell cytoskeleton known for their role in axon growth [30, 31], but also *slit*, *frazzled* and *sema-2a*, which encode proteins shown to regulate axon guidance in various systems [32, 33]. *nrg*, which encodes an L1-type cell adhesion molecule that regulates MB αβ axon branching and outgrowth [34], and *dscam*, which encodes a transmembrane protein promoting the segregation of MB αβ sister branches [35], are two other *hrp48* targets whose misregulation may explain some of the axonal defects observed in *hrp48* mutant contexts. Notably, *hrp48* loss-of-function does not affect the development of MB γ neurons, revealing that it is probably not regulating core components of the axon growth and branching machineries, but rather cell-specific regulators of axon morphogenesis.

### Sex-specific differences in MB development

MB neurons are not sexually dimorphic as γ, α’β’ and αβ neurons exhibit similar axonal projection patterns in males and females. Our study, however, revealed cryptic differences between male and female MB development as downregulation of *hrp48* induces much stronger axon overextension defects in females than in males. Such a sex-dependent sensitivity to the loss of *hrp48* function has already been reported during wing development, where Hrp48 is required to repress the expression of the female determinant *sex-lethal* (*sxl*) [23]. As shown in this study, Sxl accumulates specifically in female *hrp48* mutant imaginal disc cells and inhibits Notch signalling, thus interfering with wing growth and patterning in a sex-dependent manner. In the proposed model, Hrp48 would prevent Sxl, the female-specific determinant underlying sexual dimorphism, from disrupting the development of monomorphic tissues by limiting its expression. In MBs, however, *hrp48* mutant phenotypes do not seem to result from a misregulation of *sxl*, as i) no increase in Sxl levels was observed in *hrp48* mutant neurons, and ii) axonal projection defects were not phenocopied upon overexpression of Sxl (data not shown). We thus propose an alternative model in which the observed sex-specific penetrance of axonal projection defects would result from differential expression levels of Hrp48 target mRNAs.

Interestingly, recent high-throughput sequencing experiments have revealed that sex-specific differences in gene expression are not limited to genes involved in sexual differentiation, but are rather prevalent. Indeed, 29% of protein-coding genes have been shown to exhibit significant sex-biased expression in *Drosophila* [36], and up to 20% of *Drosophila* multitranscript genes display sex-biased expression of alternative transcripts [37]. The biological relevance of these extensive differences in gene expression largely remains to be explored; yet one can speculate that they underlie sex-specific physiological behaviors and may be exploited throughout evolution to generate sexual dimorphism.

## Supporting Information

S1 Fig Characterization of *hrp48* RNAi mutants. (A) Normalized numbers of MB neuron cell bodies in control (n = 16 MBs) and *hrp48*-RNAi (n = 10 MBs) conditions. Cell bodies were identified based on mCD8-GFP signal, and a single confocal section was used per MB (see Materials and Methods). Values were normalized to 100 for controls. ***, p<0.001 (Mann Whitney test). Precise genotypes: UAS-mCD8-GFP/+;;OK107-Gal4/+ (control); UAS-mCD8-GFP/UAS-RNAi-*hrp48* ^*#101555*^;;OK107-Gal4/+ (UAS- RNAi*-hrp48* ^*#101555*^). (B) Percentages of MBs exhibiting symmetric lobes (normal), or asymmetric or truncated lobes (projection defects) in control or UAS-RNAi-*hrp48* flies raised at 25°C (left) or 29°C (right). Numbers represent numbers of scored hemispheres. Precise genotypes: UAS-mCD8-GFP/+;;OK107-Gal4/+ (control); UAS-mCD8-GFP/UAS-RNAi-*hrp48*;;OK107-Gal4/+.
(PDF)

S2 Fig *hrp48* downregulation induces ectopic projection of both α and α’ axon branches. (A-C) MB lobes of control (A) or *hrp48*-RNAi (B, C) adult brains expressing mCD8-GFP (green), and stained with anti-Trio antibodies (red). Note that Trio localizes to both γ and α’β’ axons. Arrows in B and C point to overextended α’ axonal branches. Precise genotypes: UAS-mCD8-GFP/+;;OK107-Gal4/+ (A); UAS-mCD8-GFP/UAS-RNAi-*hrp48* ^*#101555*^;;OK107-Gal4/+ (B, C). Scale bar in A-C: 20 μm.
(PDF)

S3 Fig *hrp48* RNAi axonal projection defects in males *vs* females. Percentages of MBs exhibiting symmetric projections, asymmetric projections or truncated lobes in females (left) or males (right) expressing *hrp48*-RNAi constructs. Numbers represent numbers of scored hemispheres. Statistical comparison to the female context: *, p<0.01 (χ^2^ test). Precise genotypes: UAS-mCD8-GFP/+;;OK107-Gal4/+ (+); UAS-mCD8-GFP/UAS-RNAi-*hrp48* ^*#101555*^;;OK107-Gal4/+, and UAS-mCD8-GFP/+;UAS-RNAi-*hrp48* ^*#16041*^ */+*;OK107-Gal4/+.
(PDF)

S1 Table Phenotypic distribution of single cell α’β’ and αβ clones. Precise genotype of scored individuals: hsp-flp, UAS-mCD8-GFP/+; FRT40A *hrp48* ^*7E7-18*^/FRT40A tub-Gal80;; OK107-Gal4/+.
(XLSX)
