# Supplementary figures and images for: Drosophila Hrp48 Is Required for Mushroom Body Axon Growth, Branching and Guidance
Source: PLoS One. 2015 Aug 27;10(8):e0136610. doi: 10.1371/journal.pone.0136610 (PMC4551846; doi:10.1371/journal.pone.0136610)

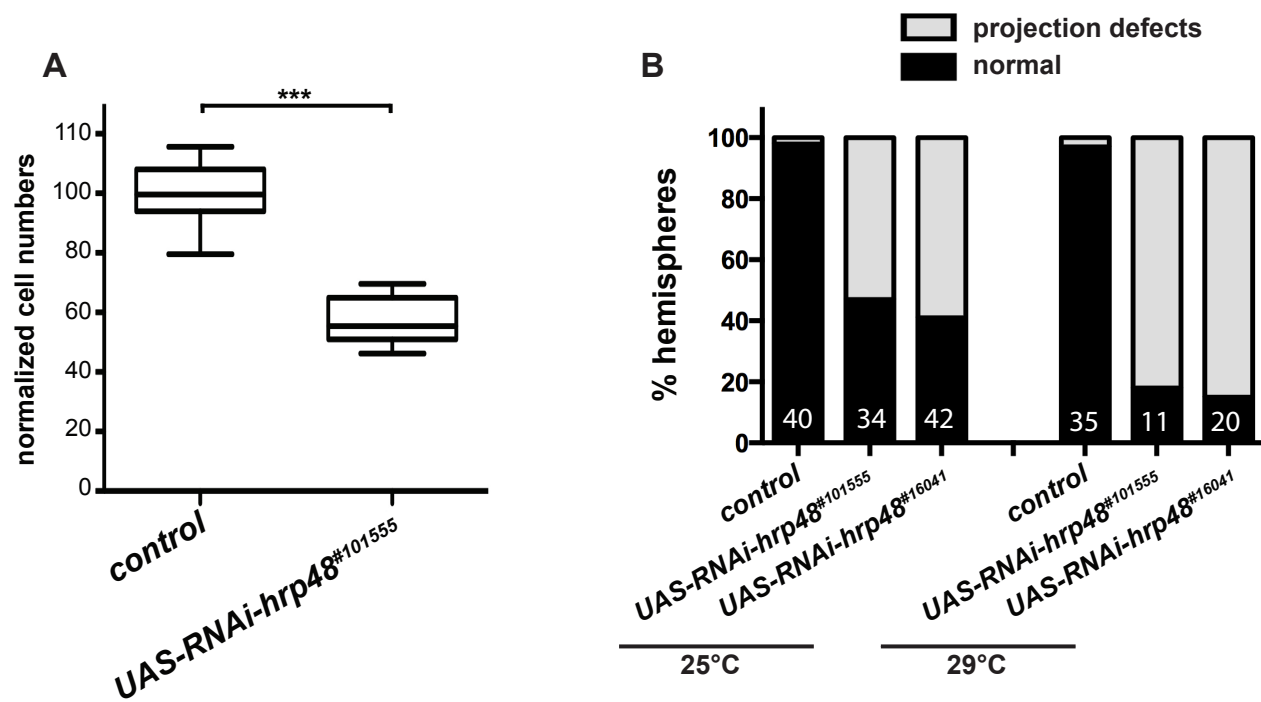

**Figure S1**

Supplement: S1 Fig — (A) Normalized numbers of MB neuron cell bodies in control (n = 16 MBs) and hrp48-RNAi (n = 10 MBs) conditions. Cell bodies were identified based on mCD8-GFP signal, and a single confocal section was used per MB (see Materials and Methods). Values were normalized to 100 for controls. ***, p<0.001 (Mann Whitney test). Precise genotypes: UAS-mCD8-GFP/+;;OK107-Gal4/+ (control); UAS-mCD8-GFP/UAS-RNAi-hrp48 #101555;;OK107-Gal4/+ (UAS- RNAi-hrp48 #101555). (B) Percentages of MBs exhibiting symmetric lobes (normal), or asymmetric or truncated lobes (projection defects) in control or UAS-RNAi-hrp48 flies raised at 25°C (left) or 29°C (right). Numbers represent numbers of scored hemispheres. Precise genotypes: UAS-mCD8-GFP/+;;OK107-Gal4/+ (control); UAS-mCD8-GFP/UAS-RNAi-hrp48;;OK107-Gal4/+. (PDF) [file pone.0136610.s001.pdf]

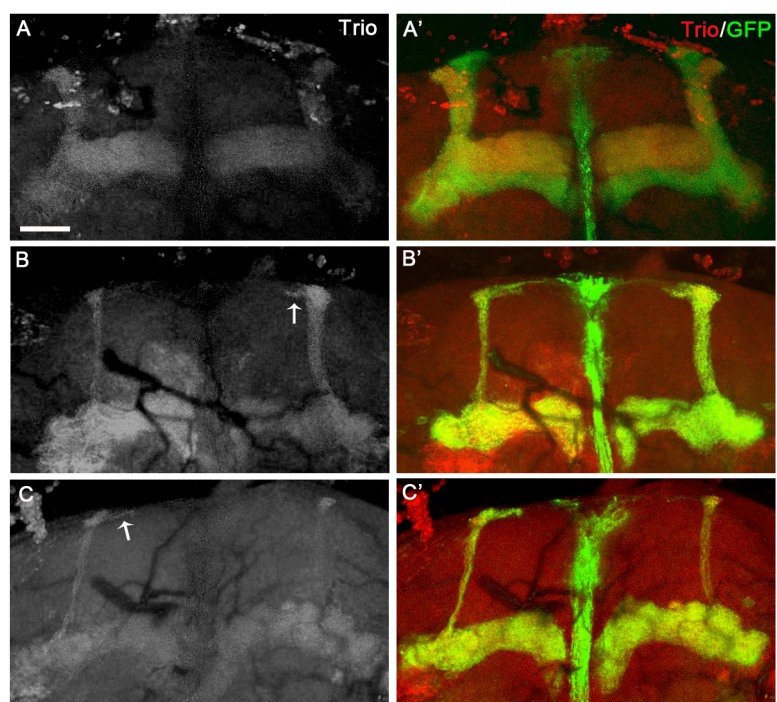

**Figure S2**

Supplement: S2 Fig — (A-C) MB lobes of control (A) or hrp48-RNAi (B, C) adult brains expressing mCD8-GFP (green), and stained with anti-Trio antibodies (red). Note that Trio localizes to both γ and α’β’ axons. Arrows in B and C point to overextended α’ axonal branches. Precise genotypes: UAS-mCD8-GFP/+;;OK107-Gal4/+ (A); UAS-mCD8-GFP/UAS-RNAi-hrp48 #101555;;OK107-Gal4/+ (B, C). Scale bar in A-C: 20 μm. (PDF) [file pone.0136610.s002.pdf]

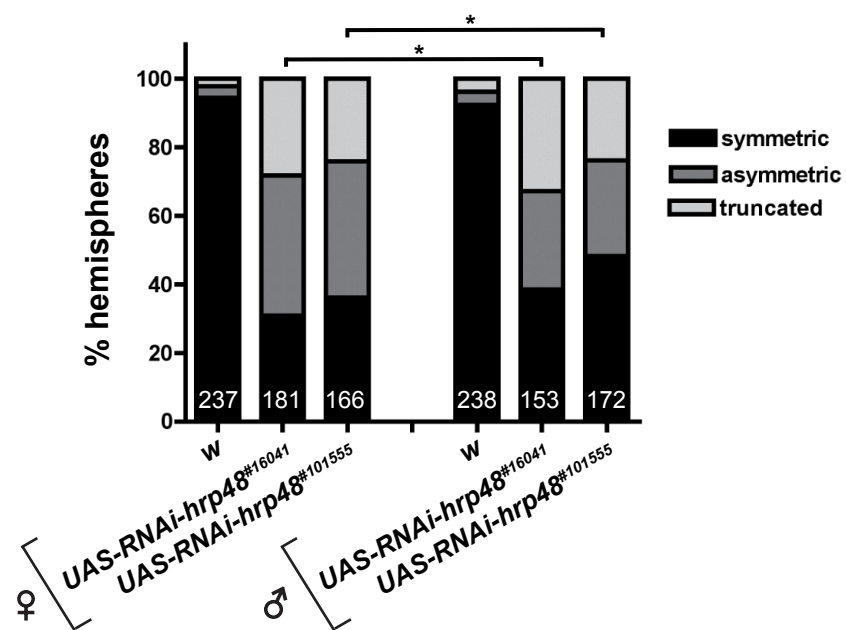

**Figure S3**

Supplement: S3 Fig — Percentages of MBs exhibiting symmetric projections, asymmetric projections or truncated lobes in females (left) or males (right) expressing hrp48-RNAi constructs. Numbers represent numbers of scored hemispheres. Statistical comparison to the female context: *, p<0.01 (χ2 test). Precise genotypes: UAS-mCD8-GFP/+;;OK107-Gal4/+ (+); UAS-mCD8-GFP/UAS-RNAi-hrp48 #101555;;OK107-Gal4/+, and UAS-mCD8-GFP/+;UAS-RNAi-hrp48 #16041 /+;OK107-Gal4/+. (PDF) [file pone.0136610.s003.pdf]
